# Virologists' Sex- and Gender-Based Medical Knowledge of COVID-19 Affects Quality of Students' Education

**DOI:** 10.1089/whr.2022.0096

**Published:** 2023-03-06

**Authors:** Helena Schluchter, Gabriele Kaczmarczyk, Ute Seeland

**Affiliations:** ^1^ Department of Anesthesiology and Intensive Care, Klinik Floridsdorf, Vienna, Austria.; ^2^ German Society for Gender-Specific Medicine, Berlin, Germany.; ^3^ German Medical Women's Association (Deutscher Ärztinnenbund), Berlin, Germany.; ^4^ Institute of Social Medicine, Epidemiology and Health Economics, Charité – Universitaetsmedizin Berlin, Berlin, Germany.

**Keywords:** COVID-19, gender, medical education, SARS-CoV-2, sex, virology

## Abstract

**Background:**

A sex- and gender-based approach to medical education is important to develop new knowledge and to improve quality of and equality within health care. Results of a systematic survey showed a lack of sex- and gender-based medical education at German medical faculties. The global severe acute respiratory syndrome coronavirus type 2 (SARS-CoV-2) pandemic is affecting people from diverse backgrounds differently, and the reciprocal interactions between biological sex and sociocultural gender aspects with regard to coronavirus disease 2019 (COVID-19) necessitate an intersectional research approach and transfer to medical education.

**Methods:**

This descriptive-phenomenological qualitative online survey focused on the sex and gender knowledge of faculty staff and the status of implementation in medical education and research at departments of virology and immunology at German university hospitals. It comprised 16 questions generated by an expert consortium based on published research data. In the fall of 2021, 36 leading virologists were invited to participate anonymously in this survey.

**Results:**

The response rate was 44%. Most experts deemed sex and gender knowledge as not that important or not important. Almost half the lecturers supported a sex- and gender-based research design and sex-disaggregated analysis of animal study data. Biological sex differences and gender aspects regarding SARS-CoV-2 were at least occasionally addressed upon a student's request.

**Conclusion:**

Virologists attributed only minor importance to sex and gender knowledge, despite scientific evidence of sex and gender differences in the field of virology, immunology, and COVID-19 in particular. This knowledge is not systematically implemented in the curriculum, but rather only occasionally passed on to medical students.

## Introduction

The national health care system should aim to continually improve the quality of and the equality within medical care. One essential innovation in medical education for reaching this goal is a sex- and gender-based approach in all disciplines. Competencies in sex, gender, and further diversity categories concerning prevention, diagnosis, and treatment of viral diseases such as severe acute respiratory syndrome coronavirus type 2 (SARS-CoV-2) are important to ensure the adequate quality of health care.

In light of the fact that mortality from coronavirus disease 2019 (COVID-19) is higher in males than in females, it is important to consider sex differences in hormonal regulation of the immune system in patients with SARS-CoV-2 infection and the interactions with their social surroundings and the environment.^1^ However, more female patients suffer from long-COVID syndrome.^2^ Sex- and gender-based research is indispensable to understand these relationships. Therefore, researchers and those responsible for higher education should be aware that studies on such viral diseases as COVID-19 must be planned, performed, and analyzed in a way that adds value to the prevention and treatment of all sexes. Moreover, additional diversity aspects, such as age and ethnicity, should be considered.

Databases on epidemiological and clinical data often lack information on sex, for example, the newly established German intensive care register for occupancy of intensive care beds.^3^ However, this information is particularly important for the adequate distribution of financial resources to establish an optimal health care structure for all sexes as well as for specific target groups.^4,5^

The recently published report by the German Federal Ministry of Health on the integration of sex, gender, and further diversity categories into curricula at medical universities and nursing and physiotherapy schools in Germany reveals considerable deficits: only 7.4% of German medical faculties offer sex- and gender-based medical education as an integral part of the curriculum.^6^ A systematic longitudinal integration into all teaching formats, including the integration into assessment, has only been achieved by 3.7% of medical universities, 2.4% of nursing schools, and 6.4% of physiotherapy schools.^6^

At the time of survey in January 2020, more than 90% of deans were aware of the importance and added value of sex- and gender-based research and education. However, 70% of them had yet to start systematically implementing sex- and gender-based learning goals in medical curricula as well as sex and gender content in assessment tools. Furthermore, sex- and gender-based teaching was not considered a criterion of faculty staff evaluation.^6^

Interestingly, according to a discipline-specific survey among medical directors of cardiology departments at German university hospitals, cardiologists include a high level of mandatory sex- and gender-based teaching and learning content in their lectures. Another discipline-specific survey among 28 clinical pharmacology faculty staff members at German university hospitals shows that more than 90% of participants are aware of sex differences in adverse drug reactions and the need for dosage adjustments with advancing age. However, for example, less than 60% of participating lecturers address these facts during their lectures concerning beta blockers, and less than 40% with regard to psychotherapeutic drugs and morphine.^6^

In light of the COVID-19 pandemic, it is important to systematically investigate the degree of sex and gender knowledge among faculty teachers for virology and immunology, responsible for research and teaching students in their fields at German medical faculties. After conducting a systematic literature review, a short poll on the knowledge of sex and gender differences in virology and immunology and their implementation in lectures was conducted in the fall of 2021.

## Methods

This descriptive-phenomenological qualitative focus survey on the knowledge of sex- and gender-based issues and the implementation in medical education and research at departments of virology and immunology at German university hospitals was conducted between October and December 2021 within a period of 6 weeks. There are 37 state and 4 private medical faculties in Germany, with 22 institutes of virology and/or immunology. Upon further online research, additional virologists were identified within the departments of microbiology, epidemiology, and infectiology. In total, 36 lecturers were invited to participate voluntary and anonymously in the online survey through the survey monkey platform (surveymonkey.com), comprising 16 questions (Supplementary Data).

The multiple choice (single answer) questions focusing on relevant sex- and gender-based issues were generated by a scientific sex- and gender-expert consortium based on a systematic literature review of publications on COVID-19 and different vaccination approaches against SARS-CoV-2 infection published between 2019 and 2022.

Descriptive statistical analysis was performed to summarize the characteristics of categorical response data, using Microsoft Excel 2019 (Version 16.41 for macOS; Microsoft Corporation, Redmond, WA).

## Results

### Participant characteristics

In total, 16 faculty teachers, of whom 2 were female, 10 were male, and 4 did not specify their sex, named virology as one of their fields of expertise. One expert named immunology, another epidemiology, and two experts named hygiene and microbiology as additional fields of expertise. One of the experts worked in virology, immunology, and hygiene and microbiology. Eight experts were responsible for both research and education, five for research only, and three participants were clinicians. The calculated response rate was 44%.

### Sex and gender in the field of virology

Most surveyed faculty teachers deemed sex and gender knowledge as not that important (12 participants) or not important (1 participant) in the field of virology. In contrast, three participants considered it very important. When asked if sex and gender content should be relevant to assessment in their field, 11 participants answered “not necessarily” and one participant selected “not required.” Four faculty teachers strongly affirmed the notion of including sex and gender content into examinations.

The fact that many genes of immune proteins are X-chromosomally located^7^ was addressed by two virologists during their lecturers and another four participants addressed this fact occasionally. Six experts did not speak about this fact during their lectures, and four did not answer the question.

**FIG. 1.**
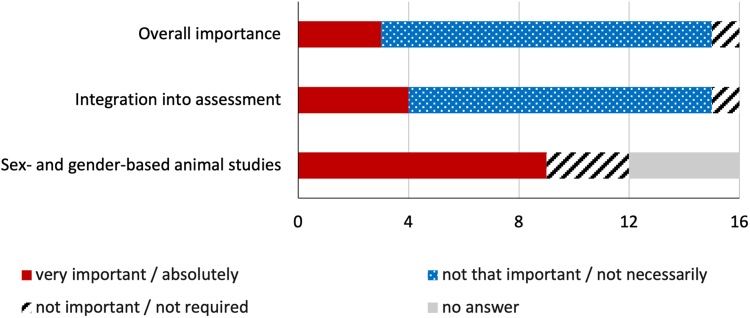
Sex and gender in the field of virology. Descriptive data, stated in absolute numbers, on the importance of sex and gender knowledge in virology and immunology, on the integration of sex- and gender-based learning goals into assessment, and on the importance of sex- and gender-disaggregated data analysis of animal studies. Only three participating virologists were convinced of the overall importance of sex and gender knowledge in the fields of virology and immunology.

A sex- and gender-based design and sex-disaggregated analysis of animal studies in infection and vaccine research were very important to nine experts, three did not consider this approach important, and four abstained from answering the question (Fig. 1).

### Sex and gender in the context of SARS-CoV-2

#### Epidemiology

Three participants did not know about sex- and age-dependent differences in the incidence of COVID-19, which have been demonstrated by national, European, and global epidemiological data sets.^1,8,9^ Nine experts were aware of the existence of these epidemiological differences and four of them were uncertain.

In a follow-up question, seven participants did not provide reasons for these observed differences in incidence. The remaining participants stated either social behaviors (*i.e.*, gender; four participants) or biological differences (*i.e.*, sex; five participants) as most likely reasons.

#### Gender

The fact that sociocultural factors function as risk modifiers for SARS-CoV-2 infection^10^ was addressed by 14 participants in their lectures (1 negation and 1 abstention). As previously mentioned, four participants traced sex differences in the incidence of SARS-CoV-2 infection most likely back to sociocultural behaviors.

#### Immune system and vaccination

Seven virologists generally broached the issue of possible sex differences in the immune response to SARS-CoV-2,^11^ and six participants at least spoke about this matter upon a student's request. Two participants did not speak about this issue at all, and one abstained from answering the question.

The higher COVID-19 mortality in males compared to females^8^ and possible reasons were addressed by 12 experts: half of them generally included this matter in their lessons, and the other half occasionally talked about it (four abstentions).

With regard to discussing sex differences in side-effects of SARS-CoV-2 vaccines, almost all participants affirmed the questions (four abstentions each). Only one virologist did not talk about myocarditis as a side-effect of messenger RNA-vaccines seen especially in young males.^12^ Four participants occasionally addressed this issue, and seven unequivocally affirmed the question.

Likewise, eight participants generally talked about cerebral venous thrombosis as a side-effect of the AstraZeneca vaccine in females^13^; the other four experts, at least occasionally, integrated this fact into their lectures.

#### Long-COVID syndrome

The fact that more female patients suffer from persisting symptoms after acute SARS-CoV-2 infection, termed long-COVID syndrome, should be of special interest for virologists.^2^ Mixed answers were given on addressing this sex difference in the incidence of long-COVID and its consideration in the evaluation of scientific publications. Seven participants addressed the issue in their lectures and/or confirmed that this sex difference should be mentioned in scientific articles about long-COVID as a criterion for high-quality publications. In contrast, five virologists negated the question (four abstentions).

#### Responsibility for sex and gender competencies

There is scientific evidence for the function of sociocultural factors as risk modifiers for SARS-CoV-2 infection.^10^ The question on a possible revision of their lectures for the purpose of including this fact was skipped by most experts (1 negation and 15 abstentions). As demonstrated by the preceding question, most of them already addressed the issue during their lectures.

Eight lecturers did not change their attitude toward sex and gender aspects on the basis of the pandemic and its impacts on society. In contrast, four participants refined their views on a sex- and gender-based approach to medicine and research, as well as on sociocultural aspects (four abstentions). Table 1 shows the sex- and gender-based lecture content in detail, already integrated into the teaching formats, not integrated, or mentioned occasionally upon a student's request.

**Table 1.** Sex- and Gender-Based Lecture Content

	Yes, ***n*** (%)	No, ***n*** (%)	Occasionally/upon request, ***n*** (%)	Abstentions, ***n*** (%)
Higher incidence of long-COVID in females	7 (44)	5 (31)	n.a.	4 (25)
Higher COVID-19 mortality in males	6 (38)	0 (0)	6 (38)	4 (25)
Sex differences in immune response to SARS-CoV-2	7 (44)	2 (13)	6 (38)	1 (6)
X-chromosomal gene localization of immune proteins	2 (13)	6 (38)	4 (25)	4 (25)
Post-mRNA-vaccination myocarditis primarily in males	7 (44)	1 (6)	4 (25)	4 (25)
Post-vector-vaccination cerebral venous thrombosis primarily in females	8 (50)	0 (0)	4 (25)	4 (25)
Sociocultural influencing factors on SARS-CoV-2 infection	14 (88)	1 (6)	n.a.	1 (6)

Do you address… descriptive data on the integration of sex- and gender-based content in virology lectures, listed by topic, stated in absolute numbers (*n*) and percentages of total answers (%). Due to rounding to full percentages, the row total can exceed 100%. Upon request = upon a student's request.

COVID-19, coronavirus disease 2019; n.a., not applicable; SARS-CoV-2, severe acute respiratory syndrome coronavirus type 2.

## Discussion

Data of this focus survey on the knowledge of sex- and gender-based issues and the implementation in medical education and research at departments of virology and immunology at German university hospitals demonstrated the lecturers' ambivalent attitude toward the topic and underlined the lack of systematic incorporation of sex and gender learning goals into the curricula. Although the participants attributed minor importance to sex and gender aspects, they incorporated sex and gender content at least occasionally into their lectures. In addition, there was support for a sex- and gender-based design of animal studies and a sex-disaggregated data analysis in infection and vaccine research.

Most virologists deemed sex and gender knowledge not that important or not important in their respective fields. This is unexpected. In contrast, more than 85% of 30 cardiologists at German university hospitals surveyed in 2020 included three or more facts about sex and gender differences into their teaching formats.^6^ The inquired issues are of great clinical importance for the adequate diagnosis and treatment of diseases, some of which have a high mortality rate. The lecturers for cardiology criticized the lack of time for teaching sex and gender aspects during their lectures due to national curricular guidelines.^6^

Besides a lack of time with regard to teaching and research, as well as curriculum development,^14^ other frequent barriers to a systematic implementation of sex and gender knowledge in medical curricula are a lack of sex- and gender-based training possibilities for lecturers and a lack of involvement of faculty staff, at least partly, due to preconceived notions toward sex and gender medicine and a lack of knowledge and ascribed importance.^15,16^ Despite this disregard among health care professionals,^17–19^ a growing number of physicians, lecturers, and medical students call for a revision of medical curricula.^20–22^ Until sex- and gender-based teaching and assessment frameworks are implemented,^23^ blended-learning didactic concepts, face-to-face interactions with online teaching methods will assist current and future medical professionals to educate themselves on sex and gender issues.^24^

Participants supported a sex- and gender-based study design and a sex-disaggregated data analysis, both required to research sex and gender differences. The selected questions for this short survey represent a fraction of the established scientific evidence of sex and gender differences in SARS-CoV-2 infection. Most of the inquired differences, if not systematically incorporated into the courses, were at least to some extent addressed by most experts during their lectures (Table 1). This raises the question why they still attributed minor importance to sex and gender knowledge in their field. Possible differences between the male and female perspective on the importance of sex and gender aspects should be taken into account.

Possibly, the predominant disregard arises from the fact that at least 10 participants were male, which is an adequate depiction of the current sex ratio in the field: female medical professionals are still underrepresented as scientists and in senior positions.^25,26^ Female health care providers and medical students consider sex and gender medicine more important than their male counterparts and they feel less prepared with regard to sex and gender competencies due to a lack of education in these skills and topics.^27–30^

Furthermore, most surveyed experts were lecturers and/or researchers. Only three participants identified themselves primarily as health care providers, more likely being confronted with sex and gender differences in their day-to-day work. In addition, the at least occasional incorporation of sex and gender content into lectures might be one reason why half the participants stated that the pandemic along with the undeniable sex and gender differences in COVID-19 did not refine their views on sex and gender aspects.

Among the surveyed virologists, nine correctly affirmed the existence of sex differences in the incidence of COVID-19 and 12 addressed the higher mortality in male patients during their lectures.^31^ In premenopausal females, SARS-CoV-2 infection rates are 15% higher compared to age-matched males, as demonstrated by real-world data assembled in 17 countries.^1^ Besides comorbidities and age, male sex is an independent risk factor for COVID-19 mortality with a 1.6- to 1.7-fold higher risk compared to female sex, shown by several studies.^8,32–35^ There might be biological, that is, hormonal and genetic, and sociocultural, that is, behavioral, underlying mechanisms of this sex- and age-specific disparity.^36–38^

Sex hormones play an important role in regulating proteins involved in SARS-CoV-2 infection and innate and adaptive immune responses.^11^ The cytokine interleukin 6 (IL-6) is downregulated by estrogens and upregulated by androgens.^39^ IL-6 is believed to be involved in the “cytokine storm” upon SARS-CoV-2 infection linked to severe forms of COVID-19 disease.^40^ Cell entry of SARS-CoV-2 depends on binding to its receptor angiotensin-converting enzyme 2 (ACE2), encoded on the X chromosome,^41^ and spike protein priming by the transmembrane protease serine subtype 2, which is a cofactor of the androgen receptor and therefore susceptible to sex hormone stimulation.^42^

In addition to ACE2, there are many X-chromosomal genes encoding proteins that function as regulators and modulators of the innate immune system. The persisting genes from the inactivated second X chromosome in females might play a protective role, as shown in better post-traumatic and post-sepsis clinical outcomes compared to males. On the other hand, this escape from X chromosome inactivation might lead to higher risk for autoimmune reactions since more females suffer from autoimmune diseases.^7^ These sex differences in genetic and hormonal regulation of the immune reaction to viral infection should be integrated as mandatory teaching and learning content into the medical curriculum. Nevertheless, the fact that many genes of immune proteins are X-chromosomally located was not systematically included in the virology lectures, and only six surveyed virologists regularly or occasionally addressed this matter.

The long-term consequences after the acute phase of the infection are the focus of current research. Some patients present with persisting unspecific symptoms, such as fatigue, concentration disorder, musculoskeletal pain, or dyspnea, even after recovery from acute COVID-19. Sex and gender differences are also apparent in a higher incidence of female patients with long-COVID syndrome. Recent studies revealed that female sex, age, and active smoking status are independent risk factors for long-COVID. The severity of acute SARS-CoV-2 infection does not correlate with the occurrence of long-COVID.^2^

Data show that it is of utmost importance to actively look for sex and gender differences not only in SARS-CoV-2 infection but also in all immunological diseases and viral infections. This systems biology approach is important to detect and manage the risk of disease of the individual patient.

Multimorbidity is associated with COVID-19 severity. Males are more likely to be diagnosed with ischemic heart disease, chronic obstructive pulmonary disease, and chronic kidney disease, their female counterparts have a higher prevalence of dementia and autoimmune disease.^43^ In addition to multimorbidity, social deprivation is a serious risk factor for COVID-19 fatality.^10,44^

Gender aspects can considerably influence the course of the disease, and their effects on exposure to, as well as transmission and progression of COVID-19 should be part of the design of preclinical and clinical studies. The knowledge gap regarding sociocultural gender effects on the trajectories and the outcome of viral diseases is significant. More efforts to close these gaps regarding gender and other diversity determinants must be made, starting with the students' education. It was therefore pleasantly surprising that 14 participants confirmed they addressed sociocultural factors as risk modifiers for SARS-CoV-2 infection^10^ and that four experts considered sociocultural behaviors as most likely reasons for sex differences in the incidence of SARS-CoV-2 infection.

### Limitations

In total, 16 faculty lecturers in the field of virology participated anonymously in this online focus survey on the knowledge and implementation of sex- and gender-based learning goals. It was noticeable that four participants skipped questions on sex- and gender-based lecture content on COVID-19. This might be due to a lack of awareness of these sex and gender differences or because these participants in general do not lecture on the respective subjects, and therefore, the questions are not applicable to their work.

Although we identified additional virologists within other departments, the total number of invited experts amounted to merely 36, since there are only 22 institutes of virology and/or immunology at German university hospitals. The results of this survey should therefore be understood as trend indicators. The multiple-choice questions generated by an expert consortium streamlined the data analysis, but did not provide the opportunity for further explanation of chosen answers. Unfortunately, only one participant took advantage of the free text option at the end of the survey. Further in-depth studies are needed to identify barriers and options for action.

The existing scientific evidence of sex and gender differences not only in cardiology but also in virology and immunology already justifies a systematic integration of specific learning goals into the teaching formats. In addition, these learning objectives should be included in the curriculum for medical students as mandatory content and should be adapted for all health professions. Nevertheless, sex and gender aspects are often inconsistently and incoherently represented in medical curricula,^45^ with subjects such as neurology, orthopedics, or immunology regularly having the least degree of implementation.^46^

Over the past years, there have been some successful curriculum re-designs at medical schools in Canada, Germany, Sweden, or the United States, which systematically integrate sex and gender medicine as a separate module as well as a cross-sectional issue into the medical curriculum.^47^ Project coordinators underline the importance of a system-level approach: sex and gender medicine should be considered mandatory for accreditation in governmental and institutional guidelines (top–down), and students, scientists, and professors should be involved in the curriculum revision process (bottom–up).^48^ Raising sex and gender awareness among all stakeholders through easy access to sex- and gender-based training and scientific evidence is of the utmost importance.^6,14,22,24,47,49^

Deans, lecturers, and authorities should emphasize the importance of an intersectional study design, sex-disaggregate data analysis, and sex- and gender-based medical education to improve public health strategies. If more experts take sex and gender differences into account, the obtained knowledge will lead to a more effective prevention of and counteraction against future pandemics.

In conclusion, sex- and gender-based research has already generated sufficient scientific evidence of sex and gender differences in virology and immunology. Faculty staff responsible for virology courses still considered sex and gender knowledge not that important or not important to their field. Presently, the education of medical students in sex and gender issues is still a matter of the individual lecturer's efforts. Broad institutional and governmental support is crucial for a systematic implementation of sex and gender content into comprehensive medical curricula.

## Supplementary Material

Supplemental data

## Abbreviations Used

ACE2: angiotensin-converting enzyme 2
COVID-19: coronavirus disease 2019
n.a.: not applicable
SARS-CoV-2: severe acute respiratory syndrome coronavirus type 2
