# Supplementary material for: Virologists' Sex- and Gender-Based Medical Knowledge of COVID-19 Affects Quality of Students' Education
Source: Womens Health Rep (New Rochelle). 2023 Mar 6;4(1):118–25. doi: 10.1089/whr.2022.0096 (PMC9989519; doi:10.1089/whr.2022.0096)
Supplement: Supplemental data [file Suppl_Data.pdf]

Deutscher Ärztinnenbund e.V.

CHARITÉ UNIVERSITÄTSMEDIZIN BERLIN

Fragebogen an alle Virologen und Virologinnen der medizinischen Fakultäten an deutschen Universitäten

Survey of virologists at medical faculties of German universities

Sehr geehrter Herr Kollege, sehr geehrte Frau Kollegin,

die Pandemie hat uns mit ungeklärten Fragen, u.a. mit zu beobachtenden Unterschieden zwischen den Geschlechtern konfrontiert. Einige wichtige Punkte sollten Einzug in die studentische Lehre halten oder werden bereits von Ihnen den Studierenden vermittelt.

Wir bitten Sie – oder eine von Ihnen bestimmte Vertretung – sich ein paar Minuten Zeit zu nehmen und 18 Fragen in einem anonymisierten Fragebogen zu beantworten.
Der Fragebogen geht an alle Virologen und Virologinnen der medizinischen Fakultäten an deutschen Universitäten.
Da wir einen aussagekräftigen Rücklauf erreichen möchten, werden wir Sie an den Fragebogen erinnern, sollten Sie bis 9. November 2021 nicht geantwortet haben. Bitte entschuldigen Sie ggf. Doppelsendungen. Man kann nur einmal antworten.

Bitte nehmen Sie sich ein paar Minuten Zeit! Vielen Dank für Ihre Unterstützung!

Dear colleague,

the pandemic has confronted us with unanswered questions, inter alia with observed sex differences. Some of those important issues should be integrated into medical education or they are already addressed during your lectures.

We kindly ask you – or a proxy of you choice – for a few minutes of your time to answer 18 questions in our anonymous survey.
The survey is addressed to all virologists at medical faculties of German universities.
Since we want to achieve a meaningful return, we will kindly remind you of our survey if you will not have participated until November 9, 2021. Please excuse multiple notifications. You can only participate once.

Please take a few minutes to answer our questions! Thank you for your support!

1. Welches sind die Schwerpunkte Ihrer Tätigkeit? [Mehrfachnennung möglich] / *What is the focus of your occupation? [multiple answers possible]*
   - ☐ Virologie / *virology*
   - ☐ Epidemiologie / *epidemiology*
   - ☐ Immunologie / *immunology*
   - ☐ Hygiene und Mikrobiologie / *hygiene and microbiology*
   - ☐ andere / *other*

2. Wo sind Sie überwiegend tätig? / *What is the predominant field of your occupation?*
   - ☐ in der Lehre / *teaching*
   - ☐ in der Forschung / *research*
   - ☐ in Lehre und Forschung etwa zu gleichen Teilen / *teaching and research in equal parts*
   - ☐ in der Krankenversorgung / *medical care*

3. Wie wichtig ist geschlechterspezifisches Wissen in Ihrem Fach? / *How important is sex and gender knowledge in your field of expertise?*
   - ☐ sehr wichtig / *very important*
   - ☐ nicht so wichtig / *not that important*
   - ☐ unwichtig / *not important*

4. Sollten geschlechterspezifische Inhalte Ihres Faches als prüfungsrelevante Lernziele im Medizinstudium berücksichtigt werden? / *Should sex and gender content be relevant to assessment in your field?*
   - ☐ unbedingt / *absolutely*
   - ☐ nicht unbedingt / *not necessarily*
   - ☐ nicht notwendig / *not required*

5. Gibt es nach Ihrer Einschätzung Unterschiede bei der Inzidenz der COVID-19-Erkrankung zwischen Männern und Frauen? / *Are there, in your opinion, differences in the incidence of COVID-19 between males and females?*
   - ☐ nein / *no*
   - ☐ ja / *yes*
   - ☐ unsicher / *uncertain*

6. Worauf führen Sie diese Unterschiede am ehesten zurück? / *To what do you most likely trace these differences?*
   - ☐ Sozialverhalten in der Pandemie ("Gender") / *social behavior during the pandemic ("gender")*
   - ☐ unzureichende Beachtung der Hygieneregeln / *insufficient compliance with hygiene rules*
   - ☐ biologische Unterschiede ("Sex") / *biological differences ("sex")*

7. Wiesen Sie bis jetzt in Ihren Lehrveranstaltungen auf mögliche Unterschiede der Immunantwort bei SARS-CoV-2-Infektion bei Männern und Frauen hin? / *Did you address possible differences in the immune response to SARS-CoV-2 infection between males and females during your lectures so far?*
   - ☐ ja, grundsätzlich / *yes, generally*
   - ☐ auf Nachfrage / *upon request*
   - ☐ nein / *no*

8. Thematisieren Sie in Ihren Lehrveranstaltungen den Zusammenhang von soziokulturellen Faktoren und dem Infektionsgeschehen? / *Do you address the connection between socio-cultural factors and the occurrence of infection during your lectures?*
   - ☐ ja / *yes*
   - ☐ nein / *no*

9. Würden Sie den Inhalt Ihrer Lehrveranstaltung entsprechend verändern? / *Would you revise the content of your lectures accordingly?*
   - ☐ ja / *yes*
   - ☐ nein / *no*
   - ☐ weiß ich noch nicht / *I don't know yet*

10. Thematisieren Sie die erhöhte Mortalität bei Männern durch COVID-19-Erkrankungen und diskutieren die möglichen Ursachen? / *Do you address the higher COVID-19 mortality in males and do you discuss possible causes?*
    - ☐ ja / *yes*
    - ☐ nein / *no*
    - ☐ gelegentlich / *occasionally*

11. Halten Sie es bei der Planung von tierexperimentellen Forschungsvorhaben zu Infektionen und Impfungen für wichtig, die Methodik und die Auswertung der Ergebnisse differenziert nach Geschlechtern anzulegen? / *Do you consider the sex- and gender-based approach to methodology and data analysis of animal studies in infection and vaccine research important?*
    - ☐ sehr wichtig / *very important*
    - ☐ nicht wichtig / *not important*

12. Weisen Sie die Studierenden darauf hin, dass ein großer Teil der Genorte, die für die Proteine des Immunsystems kodieren, auf dem X-Chromosom liegt? / *Do you address the fact that a large part of genes, encoding proteins of the immune system, is located on the X-chromosome?*
    - ☐ ja / *yes*
    - ☐ nein / *no*
    - ☐ gelegentlich / *occasionally*

13. Berichten Sie über Myokarditiden nach mRNA-Impfungen vorwiegend bei jüngeren Männern und diskutieren mögliche Ursachen? / *Do you address the fact that myocarditis after mRNA-vaccinations is primarily seen in young males and do you discuss possible causes?*
    - ☐ ja / *yes*
    - ☐ nein / *no*
    - ☐ gelegentlich / *occasionally*

14. Thematisieren Sie Hirnvenenthrombosen nach Impfung mit AstraZeneca-Impfstoff vorwiegend bei Frauen und diskutieren mögliche Ursachen? / *Do you address the fact that cerebral venous thrombosis after vaccination with the AstraZeneca vaccine is primarily seen in females and do you discuss possible causes?*
    - ☐ ja / *yes*
    - ☐ nein / *no*
    - ☐ gelegentlich / *occasionally*

15. Hat die Pandemie mit ihren Auswirkungen auf die Gesellschaft Ihren geschlechtersensiblen Blick geschärft? / *Did the pandemic and its impact on society refine your sex and gender view?*
    - ☐ ja, vor allem in der Medizin und in der Forschung / *yes, above all in medicine and research*
    - ☐ ja, vor allem im soziokulturellen Bereich / *yes, above all in socio-cultural aspects*
    - ☐ in beiden Bereichen / *in both areas*
    - ☐ in keinem von beiden / *in neither*

16. Das Long-COVID-Syndrom tritt nach heutigen Kenntnissen häufiger bei Frauen als bei Männern auf. Berücksichtigen Sie dies in Lehrveranstaltungen oder bei der Beurteilung der Güte von Publikationen zu diesem Thema? / *According to current knowledge, long-COVID syndrome is more common in females than in males. Do you take that into account during your lectures or in the evaluation of the quality of publications on this subject?*
    - ☐ ja / *yes*
    - ☐ nein / *no*

17. Sind Sie / *You are*
    - ☐ männlich / *male*
    - ☐ weiblich / *female*

18. Möchten Sie uns etwas in freier Textform mitteilen? / *Do you want to communicate something in free text form?*

